# Investigation of Tactile Sensory System Configuration for Construction Hazard Perception

**DOI:** 10.3390/s19112527

**Published:** 2019-06-03

**Authors:** Sayan Sakhakarmi, JeeWoong Park

**Affiliations:** Department of Civil and Environmental Engineering and Construction, the University of Nevada, Las Vegas, NV 89154, USA; sayan.sakhakarmi@unlv.edu

**Keywords:** tactile system, vibration, message delivery, construction safety, hazard perception

## Abstract

The application of tactile-based wearable devices to assist in navigation for people with low sight/low memory has demonstrated the feasibility of using such devices as a means of communication. Accordingly, a previous study in construction research investigated various parameters of tactile signals to develop a communicable system for potential application in construction hazard communication. However, the nature of construction limits the application of such devices to the body of construction workers, and it is important to understand sensor design parameters for improved communication, which has not been given significant attention yet. Therefore, this study aims to determine key design factors such as the number of motors, spacing between sensors and the layout of a tactile sensory system to be used for communicating construction hazards to workers. For this purpose, this study focused on identifying the number of motors based on extensive literature and the problem of construction safety as to hazard communication, determining the arrangement that allowed for effective delivery and perception of information with minimum effort. The researchers conducted two experimental studies: First, to determine the minimum spacing between vibration motors that allows for the identification of each individual motor with high accuracy; and second, to determine the layout of motors that is suitable for effective communication of multiple types of information. More importantly, the tactile-sensor configuration identified from this study allows the workers to learn the signal patterns easily in order to identify multiple types of information related to hazards. Using such a communication system on construction sites will assist in transmitting hazard-related information to workers, and thus, protect the lives of workers. Such wearable technologies enable the detection of individual-level hazards and prevent worker fatalities and severe injuries.

## 1. Introduction

### 1.1. Background

The Bureau of Labor Statistics records indicate an increasing trend in worker fatalities since 2009 [1]. Among the total worker fatalities in the US, the construction industry alone contributes to almost 20% of fatalities in private industry [2]. Approximately 60% of such fatalities in 2017 resulted from falls, strikes by objects, and electrocutions, as well as workers being caught in between objects (e.g., equipment); these are also known as the fatal four in construction [2]. Further, transportation accidents accounted for 40% of the total worker fatalities in the same year [1]. These statistics indicate that fatal accidents are common in workplaces, and the passive safety measures adopted during construction are insufficient to prevent the consequences of such accidents. Further, studies have found that construction superintendents are not able to identify all possible hazards in work zones [3,4,5,6]. As such, the researchers have determined the necessity of a supplementary system capable of identifying possible hazards in active work zones. Such a system acts as an additional sense for the construction personnel, and assists in minimizing fatal risks by alerting workers of these possible risks. On this account, researchers have explored the applicability of real-time hazard detection systems for preventing fatalities and severe injuries.

Studies to develop real-time hazard detection systems have used a wide variety of sensing technologies. One of the frequently used sensing technologies is proximity detecting sensors [7,8,9,10,11,12,13,14,15], which prevent construction workers, drivers, equipment operators, etc., from proximity hazards by generating real-time alerts. Various studies to solve proximity issues on job sites have utilized different technologies, such as radio frequency identification (RFID) [8,11], ultra-wideband (UWB) [12,16], global positioning system (GPS) [10], inertial measurement unit (IMU) sensors [10], bluetooth low energy (BLE) based sensors [7], unmanned aerial vehicles (UAV) [17], etc. These studies [8,9,10] have demonstrated the capabilities of their systems to minimize proximity hazards on construction sites, mainly the fatalities and severe injuries resulting from workers being struck by vehicles or equipment. Besides proximity issues, researchers have also attempted to resolve other types of hazard on construction sites, such as the use of sensor data to automate the monitoring of temporary structures [18,19] and prevent fall hazards [20]. There are many studies that have used computer vision techniques to identify the unsafe behavior of workers [21] and failure of temporary structures [22,23].

Most of these automated approaches focus on improving the overall safety in construction work zones. However, these studies do not address the individualized safety issues, which are more important when attempting to prevent individual worker fatalities. Thus, researchers are now interested in developing systems that are capable of generating individualized information. Accordingly, researchers have developed various wearable technologies to resolve construction safety issues. In recent years, there has been a significant increase in studies to explore the application of wearable devices in construction. One of the most studied topics is the application of wearable devices to identify unsafe worker positions [24,25,26,27,28,29,30,31]. The wearable devices monitor the posture and body movement of workers while they are performing various construction activities and unsafe positions are reported back to them. Accordingly, workers correct their positions and prevent themselves from fatalities or injuries resulting from such postures. For this purpose, researchers have mostly used IMU-based devices [25,32,33], pressure sensors [30], and built-in smartphone sensors [26,27,31]. Other studies have used wearable devices to detect physical fatigue among workers [34,35,36], as well as the psychological status of workers [36,37] in order to prevent accidents due to unsafe worker behaviors. Researchers have also used insole pressure sensors to detect potential worker instability [38], and thus, prevent fall hazards.

The studies with wearable technologies have mainly focused on preventing hazards related to unsafe worker behaviors on construction sites. However, there are limited studies on using wearable technologies to warn workers of potential hazards surrounding them. An augmented reality-based wearable device proposed by Kim et al. [39] has the capability of alerting workers to prevent possible hazards. Their system warns workers of the orientations and distances of potential hazards, along with indicating the level of safety for individual workers. However, the system is designed to generate warnings based on the visuals of workers. Thus, it may not be able to prevent worker injury due to possible hazards beyond their vision. Despite the high potential of their system to alert construction workers, limited sight, due to obstructions or low vision, and limited hearing, due to loud noises on construction sites, may be limitations as well. As such, it is not effective to only depend on the senses of workers to prevent construction accidents; a few recent studies found that visual and auditory alerts often fail to alert workers [40,41]. Studies have shown that additional sensory systems can play a significant role to supplement these limited senses [42,43]. Therefore, this study focuses on exploring the use of a wearable system that activates the sense of touch in workers. For this purpose, the researchers developed a tactile signal-based wearable system to inform workers of detected hazards through vibration signals on their backs.

### 1.2. Research with Tactile Sensors

While exploring studies utilizing tactile sensors, it has been observed that most of these studies focused on assisting people for navigation purposes. Van Erp [44] investigated the effectiveness of perceiving tactile directional information using 15 tactors on a waist belt around the torso of static test participants. Other studies [45,46,47] have demonstrated the application of such a tactile system on rugged terrain for military usage and concluded that the tactile-based navigation system is capable of guiding effectively during local navigation. Grierson et al. [48,49] tested a wearable belt with four vibrating motors to facilitate the navigation of older people who have limited visual capability or low memory. The vibration motors are fixed on a belt in such a way that they lie at the cardinal front, back, left, and right parts of the body. The system is integrated with a GPS for location tracking, and it demonstrated the capability to guide people to their pre-set destinations by using vibration signals to specify direction and distance. However, this study was limited to testing four obvious directions: front, back, left, and right, which might have resulted in better performance of the system. Other studies [50,51,52] demonstrated the capability of a system with eight vibration motors equally spaced around the waist, for assisting the navigation of visually impaired people. Marston et al. [53,54] demonstrated the applicability of a vibrotactile system wearable on the wrist to assist navigation of visually impaired individuals. The system is integrated with a GPS and it uses a single vibrator to indicate correct heading direction of the person wearing it. Thus, the problem with this system is that, at turning points, a person has to spend some time turning around to determine the correct direction to move forward. Furthermore, researchers have also developed a wearable system capable of transforming visual information captured with a camera into tactile signals to assist in navigation [55].

While observing the interface of vibration motors used in the previously discussed navigation assistance systems, it was found that most of these studies [44,45,48,49,50,51,52] had their motors arranged around a waist belt at an equal spacing to assist in navigation. Few studies [56,57] used a 3 × 3 back-array arrangement of motors on the back of people to guide in navigation. These systems with a back array layout of motors created sequential vibrations of motors to indicate direction to users. Srikulwong and O’Neill [58] compared the effectiveness of transmitting directional information using three different interfaces of vibration motors: 3 × 3 back-array at 50 mm and 80 mm of spacing between motors, and eight motors arranged around a waist-belt. The study identified the waist-belt interface to be the most effective for navigation purposes, while the interface with the 3 × 3 back-array at 50 mm spacing had the worst performance. The researchers also concluded that the directional information could be effectively delivered with vibration motors arranged around the body to resemble actual directions in the horizontal plane. With such an arrangement, it is easier for users to identify directions based on the location of a single vibrating motor around their body.

Apart from the studies to determine the feasibility of using a vibration-based system for navigation purposes, Durá-Gil et al. [50] investigated different vibration patterns for effective directional information. Among the eight vibration motors placed at equal spacing around the waist, five motors at the front were used for indicating direction, such as continue straight and turn left/right at different angles. Other motors at the back were used to indicate that the users should stop or slow down. The researchers conducted a pairwise comparison to identify a suitable vibration pattern from a set of different vibration patterns for particular directional information. Their study concluded that vibrations with single bursts are effective to deliver directional information, compared to vibrations in sequence.

It is evident that researchers have achieved significant progress in developing tactile sensor-based navigation devices mainly to help people requiring assistance to navigate around freely. These developments have demonstrated the feasibility of using such a tactile-based application to enhance the sensing capability of construction workers to detect surrounding hazards. In response to the problem of construction safety, Cho and Park [59] developed a prototype tactile sensory system and investigated signal parameters in hope to provide an artificial sensing ability to improve the perception capability of workers. Their study mainly focused on investigating three basic signal parameters—signal intensity, signal duration, and duration between signals—to build basic language parameters for construction safety applications. The researchers identified eight distinct signal units based on those signal parameters, for effective communication with construction workers. However, their study did not account for system design factors that can help effective construction of messages that comprise hazard information. In order to address this issue, this study focuses on determining the configuration of a tactile sensory communication system for the perception of hazard information.

## 2. Research Objective and Scope

This study aims to investigate design parameters and determine the configuration of a tactile sensory system best suited for communicating information related to potential construction hazards to workers in an active work zone. For effective communication with the system, the tactile signals from individual vibration motors must be easily distinguishable and meaningful. Thus, the researchers have designed this study to achieve three specific objectives:

1. To determine the number of vibration motors to be used in the system to effectively deliver hazard information.
2. To determine the optimum spacing between the vibration sensors in both the horizontal and vertical axes; such spacing will result in a higher accuracy of detecting signals from consecutive sensors.
3. To determine an arrangement of vibration motors in such a way that the configuration is capable of transmitting easily distinguishable and meaningful signals with higher perception accuracy.

This study is part of an effort to develop a wearable system capable of informing individual workers of potential risks in their surroundings using tactile signals. Several studies have demonstrated that potential risks at work zones can be identified using various sensing technologies. For example, proximity sensing technologies [7,8,12,13,14,15] are capable of quantifying hazard information in terms of the distance or direction of the potential hazard location to prevent accidents. Similarly, Park et al. [60] demonstrated the quantification of construction safety hazards using tracking sensors. With the availability of such hazard detection and quantification systems, information suitable to define construction hazards can be easily acquired. Using the wearable tactile communication system discussed in this study, such hazard information can be promptly transmitted to workers. Thus, for the reliable transmission of warning messages, the researchers focus on identifying the optimal configuration of vibration motors in this study. The vibration motors used in this study are embedded on a waist belt, and this wearable system is designed to be worn on a worker’s back. The reason for selecting the back of workers is that this area is generally free while working, compared to other parts of the body. Further, using vibration motors on the back of workers provides a larger open space to arrange the motors with greater flexibility. Previous studies using tactile sensing for navigation purpose identified the arrangement of motors around the waist to be effective, compared to the back-array arrangement of motors [58]. However, this study intends to communicate multiple types of information at a time, for which the arrangement of motors around the waist would not be effective. Thus, this study adopted a back-array arrangement of motors.

## 3. Materials and Methods

**Figure 1:**
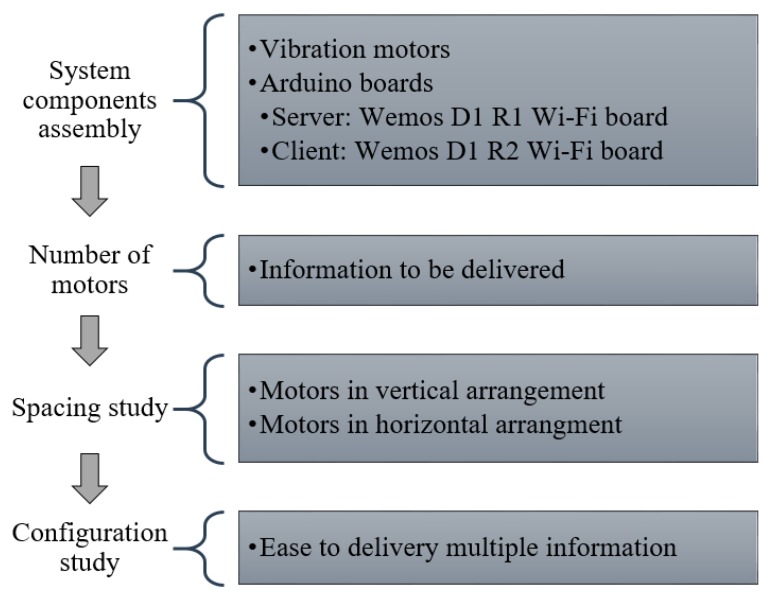
General approach.

The general approach of this study (Figure 1) is to determine the best layout of vibration motors by locating the motors at suitable spacing distances and giving a configuration, such that the layout is capable of delivering multiple types of information without difficulty. Therefore, the first step in this study was to determine the number of vibration motors required to deliver hazard information to workers. Then, to determine the motor configuration, the researchers conducted two experimental studies. The first was to determine the minimum spacing between the motors beyond which it is difficult to identify the signals from adjacent motors distinctly. The next was to test different motor layouts to identify the best one. The detailed methodology of this experimental study is explained in the following sections.

### 3.1. Assembly of System Components

**Figure 2:**
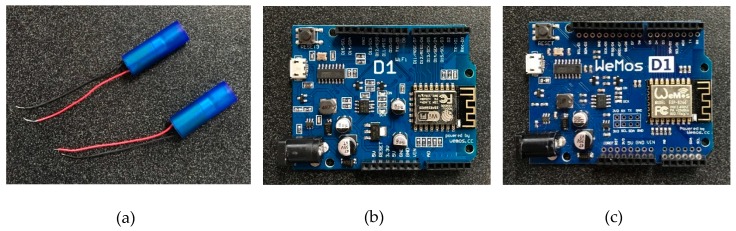
System components: (**a**) Vibration motors; (**b**) server; (**c**) client.

The major components used in this tactile sensory system included the cylindrical vibration motors, a Wi-Fi enabled Wemos D1 R1 board acting as the main server of the system, and a Wemos D1 R2 board acting as the client, which was responsible for triggering the vibration motors upon receiving commands (Figure 2). 

The vibration motors shown in Figure 2a have a rated voltage of 3.0 Volt, and a rated speed of 12,000 ± 2500 rpm. Figure 2b,c shows two types of Wemos boards used as a server and a client, respectively. The client board has vibration motors connected with it, and it triggers the vibration motors. This sensor system is firmly attached to a waist belt. The server board wirelessly links the client board with an Android mobile device, which is used to deliver signals to the system manually. In this study, pre-defined signal information was used to trigger the vibration motors.

### 3.2. Number of Vibration Motors

This study considered the types of information intended to be delivered to construction workers as the factor to determine the required number of motors. As the system is intended to inform the workers of potential hazards, the authors identified the signals with information on potential hazard location and type to be meaningful, as considered by other researchers [39]. The effective transmission of such signals to workers prepares them for necessary preventive actions.

**Figure 3:**
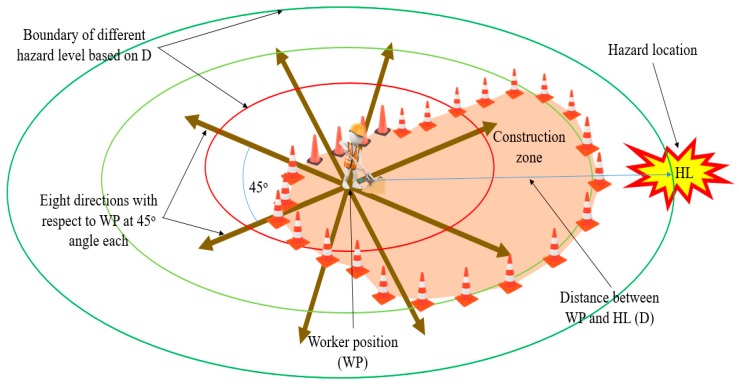
Hazard location information.

This study considered two major factors to identify hazard location (HL) with reference to individual worker position (WP) in the construction zone. The first is the direction of HL with respect to WP. This study considered eight directions (as was used in a study [15]), each at 45° with respect to WP in the horizontal plane. Next is the distance of the worker from the HL: The lower the distance between the WP and HL, the higher the level of hazard to workers. Accordingly, the distance between the WP and HL was differentiated into different regions to reflect the hazard level. Therefore, the vibration signals include information related to HL for workers instead of exact distance information. Figure 3 illustrates the HL information. 

Previous studies have demonstrated the effectiveness of mapping motor locations corresponding to directional information [58]. Thus, the authors selected eight motors to represent eight directions individually. An additional two motors were selected to transmit information related to hazard level and hazard type. Therefore, the tactile sensory system configuration uses ten vibration motors together with Arduino boards that can wirelessly transmit signals through Wi-Fi connectivity. 

### 3.3. Experimental Study

**Figure 4:**
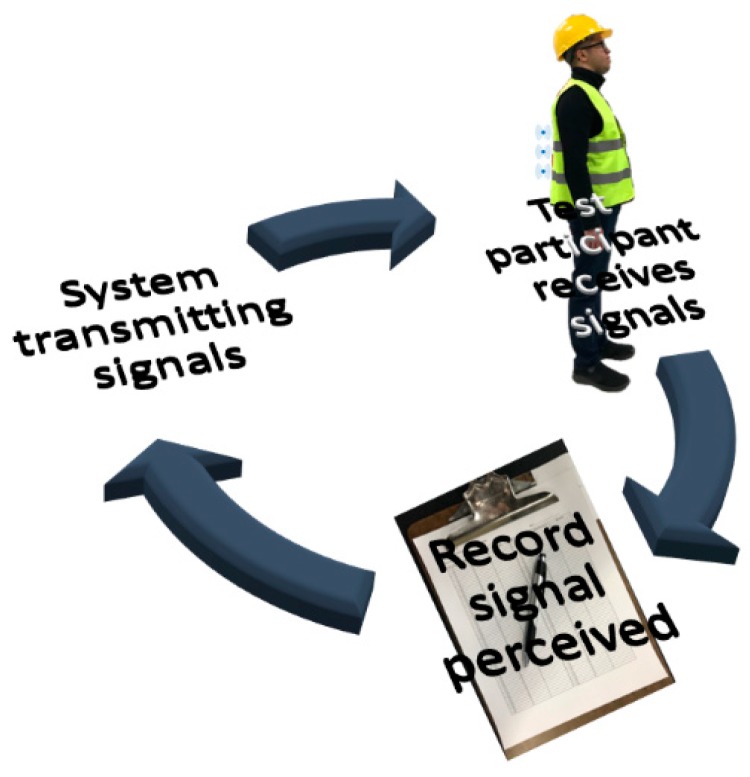
General experimental procedure.

This experimental study was divided into two parts. The first study dealt with identifying the optimum spacing between adjacent vibration sensors. Results from this study were used in the second study to configure the ten vibration motors so that individual vibration signals can be recognized with ease and greater accuracy. Figure 4 shows the general experimental procedure in which vibration signals were transmitted to test participants wearing a waist belt with vibration motors, and the participants were required to identify the signals and inform verbally. The messages sent to the participants and the messages identified by them were recorded for analysis. Before actual testing, the test participants were trained to learn signals from different vibration motors. The detailed experimental procedures for the two studies are explained in the following sections.

#### 3.3.1. Spacing of Vibration Sensors

This part of the experimental study focuses on identifying the optimum spacing between adjacent vibration motors. Vibration motors placed at a proper spacing allowed workers to identify signals from individual motors without any difficulty. When the motors are placed too close to one another, it is difficult to identify individual signals. Thus, for the optimal perception of individual vibratory signals, the spacing between sensors must be adjusted to avoid confusion between signals from two adjacent motors, as much as possible. The ability to correctly identify individual vibration motors enables the delivery of specific information using signals from a combination of different motors. 

**Figure 5:**
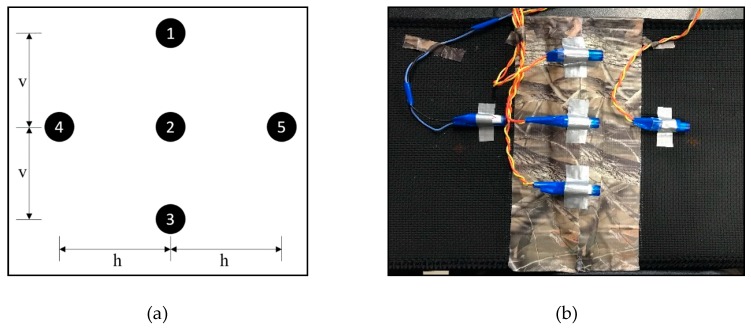
General arrangement of vibration motors for spacing study: (**a**) theoretical arrangement of vibration motors; (**b**) vibration motors embedded on waist belt.

This study used five vibration motors for the spacing study. These motors were arranged with different spacing in vertical (v) and horizontal (h) directions, as shown in Figure 5a. Figure 5b shows the vibration motors embedded on a waist belt for the experimentation purpose. For the motors arranged in the vertical direction, the width of the waist belt was a limitation. Thus, the authors conducted preliminary tests to make sure that the testing for motor spacing using a waist belt of width 8.5 inches is reasonable. From the preliminary test results, it was determined that the vibrations from the motors were accurately identifiable at a spacing up to 3.25 inches between motors. Thus, for our experimentation purpose, three vibration motors (1, 2, and 3) were arranged at a maximum spacing of 3.25 inches, leaving one inch on both edges of the waist belt. For the motors in the horizontal direction, there is no such limitation in terms of the size of the waist belt used. However, the same spacing of 3.25 inches was used for the first set of testing. For the subsequent spacing, the distance between two motors was reduced by 0.25 inches each time. However, due to the alignment of the cylindrical motors in the horizontal direction, as shown in Figure 5b, a minimum distance of 2.50 inches was used as horizontal spacing. This study tested six different spacing configurations, as shown in Table 1. 

#### 3.3.2. Configuration of Vibratory Sensors on Waist belt

**Figure 6:**
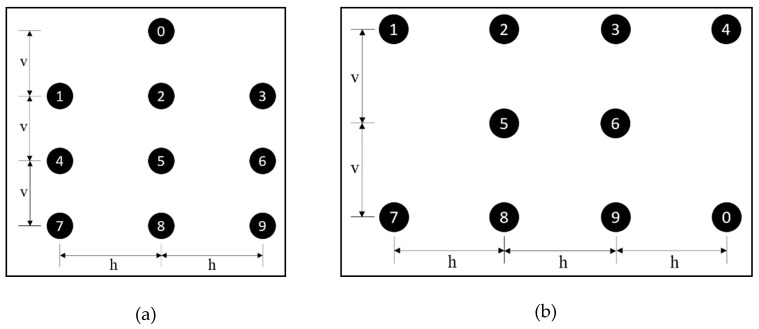
Configuration of vibration motors: (**a**) 1-3-3-3 configuration; (**b**) 4-2-4 configuration.

This experimental study considered a symmetrical layout of motors about the spinal line on the back to make it easier for the workers to learn the signal patterns delivering multiple types of information by triggering multiple motors in a sequence. Therefore, this study considered a symmetrical back-array arrangement of vibration motors to be effective for efficient communication. Accordingly, this study identified two potential layouts for the motors: a 1-3-3-3 configuration, which has one motor on the first row and 3 motors on each of the next three other rows as shown in Figure 6a, and a 4-2-4 configuration, which has four motors on the first row, two motors on the second row and four motors on the third row as shown in Figure 6b. 

The 1-3-3-3 configuration was obtained by using an additional motor on a 3 × 3 array system that was used by previous studies [56,57]. In this configuration, four motors, 0, 2, 5, and 8, lie along the spinal line. This configuration is designed to transmit eight types of directional information using motors 1, 2, 3, 4, 6, 7, 8, and 9, as these motors are located in the layout to resemble eight directions to users. The other motors, 0 and 5, are intended to deliver information on hazard level and hazard type. Similarly, the 4-2-4 configuration is symmetrical about the spinal line with five motors on each side of the spinal line. This layout uses motors 1, 2, 3, 4, 7, 8, 9, and 0 to deliver directional information. Motors 5 and 6 are used to provide information on hazard level and hazard type. Such assignment of individual motors for different information allows the workers to perceive transmitted information more accurately. The configuration study was conducted in two sequential tests as follows:

##### Preliminary Test

Initially, a preliminary test was conducted to determine the best configuration between the two layouts, as shown in Figure 6. For this purpose, the test participants were asked to identify signals from different motors from both the configurations. Based on the recorded data, the accuracies of correctly identifying the signals from individual motors on both motor configurations were computed. Then, the motor configuration with higher accuracy was selected for additional tests. 

##### Follow-Up Test on Selected Configuration

The main purpose of the follow-up test was to validate the results from the preliminary tests. Thus, the configuration identified to be suitable could be tested further with new test participants who have not participated in the previous tests under this study. Such a validation would ensure the applicability of the system design for communication purposes. 

## 4. Experimental Study Results

### 4.1. Spacing Study Results

The spacing study was conducted with six test participants; each was capable of detecting vibration signals on their back. For each set of spacing, all test participants were first trained to identify the individual vibration motors with vibration signals from all five motors on their back. The vibration motors were triggered for a duration of 250 milliseconds. Then, they were asked to identify 60 random signals from each of the five motors. The authors recorded these signals transmitted to the test participants and their responses. This study collected a total of 10,800 signals of data (i.e., 60 signals × 5 motor × 6 test participants × 6 spacing) for the six spacing configurations. Table 2 summarizes the perception accuracy of all of the test participants for the individual motors at each spacing.

The table shows a decrease in accuracy of identifying individual motors with the decrease in spacing between motors, that is, from 99.89% at the first spacing to 95.50% at the last spacing. These results show that the maximum spacing of 3.25 inches selected for the spacing study is reasonable. It is also evident that the decrease in accuracy is mainly impacted by the incorrect identification of motors arranged on the vertical axis: M1, M2, and M3. The table clearly shows a decreasing trend in accurately identifying motors arranged in the vertical axis as the spacing decreases. However, such a trend is not observed when the spacing of motors arranged in horizontal axis is decreased. Additionally, M1 is observed to be the least accurately perceived among these three motors. At the spacing of 2.5 inches, the accuracy of identifying signals from each motor is approximately 95%. Thus, it is suitable to use a minimum spacing of 2.5 inches between the motors.

Table 3 and Table 4 illustrate the separate summary of test results for signals from motors arranged on the vertical and horizontal axes, respectively. These results show that there are more incorrect identifications of motors arranged on the vertical axis (i.e., M1, M2, and M3) compared to the motors arranged on the horizontal axis (i.e., M4, M2, and M5). The participants incorrectly perceived 5.83% of signals from Motor 1 as the signals from Motor 2. Similarly, approximately 1% of the signals from Motor 2 were misperceived as Motor 1 and Motor 3, and 2.36% of the signals from Motor 3 were misperceived as coming from Motor 2. All test participants correctly identified most of the signals from the motors arranged on the horizontal axis, with less than 0.20% incorrect perceptions.

### 4.2. Configuration Study

#### 4.2.1. Preliminary Test Results

**Figure 7:**
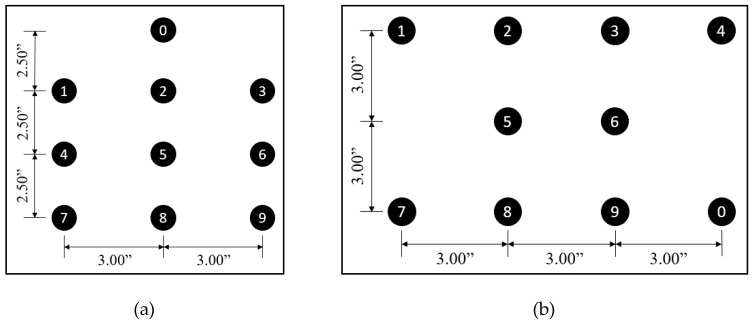
Spacing of vibration motors for preliminary test: (**a**) 1-3-3-3 configuration; (**b**) 4-2-4 configuration.

The preliminary configuration test was conducted to determine the configuration that is effective for producing the accurate identification of individual motor signals among the two vibration motor layouts, as shown in Figure 6. For this purpose, the researchers first configured the ten vibration motors for both layouts using a minimum spacing of 2.5 inches. In the case of the 1-3-3-3 layout, the spacing of 2.5 inches was used between motors arranged on the vertical axis, while a spacing of 3 inches was used for the 4-2-4 layout. In both layouts, motors were arranged on the horizontal axis at a spacing of 3 inches. Figure 7 shows both configurations with the spacing used.

**Figure 8:**
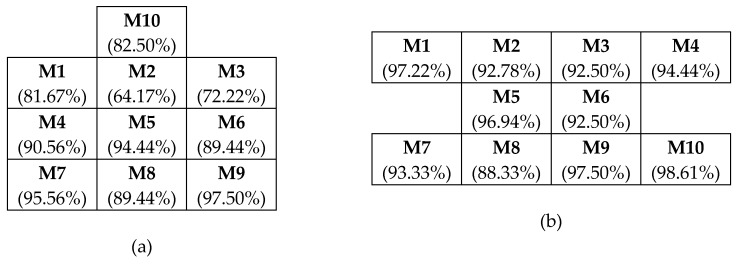
Accuracy of identifying individual motor signals: (**a**) 1-3-3-3 configuration; (**b**) 4-2-4 configuration.

There were six test participants involved in the preliminary study. All participants were trained to identify signals from individual vibration motors before the actual tests. Then, each participant was asked to identify 120 random signals from 10 different vibration motors. Each test lasted for 15 min. For each participant, the test was repeated five times at an interval of five minutes between the tests. Thus, the researchers recorded a total of 3600 sets of data (i.e., 6 participants × 5 tests × 120 signals) for each of the configurations separately. The test participants expressed having difficulty in identifying signals from the 1-3-3-3 configuration compared to the 4-2-4 configuration. Table 5 shows the overall summary of the preliminary test based on all collected data. Figure 8 shows the overall accuracy of the identification of individual motors in both configurations.

Based on these comparison results from Table 5 and Figure 8, it is evident that it was more difficult for the test participants to identify individual motors in the 1-3-3-3 configuration than the 4-2-4 configuration. In the case of the 4-2-4 configuration, such difficulty was small, with a minimum accuracy of 88.33% and an average accuracy of 94.42% to identify individual motors, compared to 64.17% and 85.75% in the case of 1-3-3-3 configuration. Following these results, the 4-2-4 configuration was selected for further data collection to confirm that it is best suited for the intended purpose of communication. Note that this signal identification is based on a learning process where people perform better with continuous use. At this stage, minimal training was given to participants for identification of an effective configuration. Given this, the identified configuration offers better potential to facilitate future use of the tactile hazard perception system.

#### 4.2.2. Follow-Up Test Results

**Figure 9:**
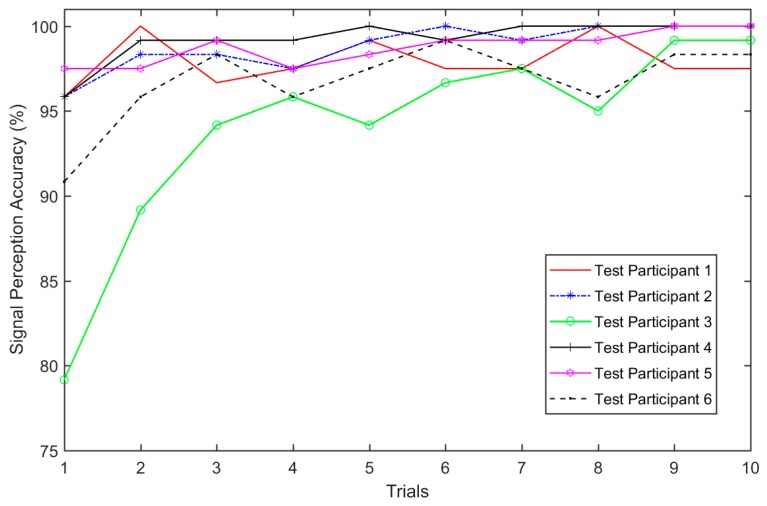
Individual signal perception accuracy.

For the follow-up data collection with the 4-2-4 configuration, there were six test participants who did not participate in the previous tests. All participants were trained to identify signals from all motors before starting the test. Then, each test participant was asked to identify 120 random signals from 10 different vibration motors. This test was repeated ten times for each test participant, and each test lasted for 15 min. The researchers recorded 7200 responses (i.e., 120 signals × 10 tests × 6 participants) from all test participants. Figure 9 shows the individual motor signal perception accuracy of all test participants for the 10 trials. The plot shows an improvement in signal perception accuracy for all test participants over the 10 trials with average perception accuracies of 97.92%, 98.83%, 94.00%, 99.25%, 98.75%, and 96.75%, respectively, for the six test participants. 

Combining data from all participants, it was determined that the overall accuracy of identifying signals correctly is 97.58%. Most of the incorrect responses were between the motors arranged on the vertical axis of 2, 5, and 8, and between motors 3, 6, and 9. These inaccuracies accounted for over 80% of the incorrect identifications. Table 6 shows the accuracy of identifying individual motors.

In this experimental study, the outcome of the responses from the participants are either correct or incorrect. As such, this experiment reflects the Bernoulli trial [61]. Thus, to check the statistical significance of the test results, the researchers computed the 95% confidence interval for the probability of success (i.e., correct identification) as follows:

Total number of trials (n) =7200

Total number of success (X)=7026

$$probability\;of\;success\;\left(p\right)\;=\frac{X}{n}=0.9758$$

$$95\%\;Confidence\;Interval\;=p\pm 1.96\;\times \;\sqrt{\frac{p\left(1-p\right)}{n}}$$

∴ 95% Confidence Interval =(0.9723,0.9794)

As the probability of correctly identifying signals from individual motors lies within a 95% confidence interval, these test results are valid. Therefore, these results show that the 4-2-4 configuration of vibration motors at the spacing of 3 inches can be effectively used for the intended purpose of communicating multiple types of information.

## 5. Discussion

The higher misperception of signals from motors arranged in the vertical axis compared to those arranged in horizontal axis indicates that it is difficult to perceive signals from motors arranged vertically. Such difficulty exists even when the motors are arranged at a larger spacing, and it is one of the reasons for the unfeasibility of the 1-3-3-3 configuration of ten motors compared to the 4-2-4 configuration, for the intended purpose of this study. 

During the experimental study, it was noticed that different factors affect the performance of test participants to identify tactile signals, such as the position of the waist belt and the contact of the vibration motors on the backs of the test participants. To avoid the impact of such factors, it was confirmed that the waist belt was used on top of a thin inner cloth and well positioned around the body. It was also identified that the rated speed of the vibration motor used in this study was another factor responsible for some inaccuracies. As mentioned in the previous sections, the vibration motors have a rated speed of 12,000 ± 2500 rpm, which is ±20.83% deviation in the vibration intensity. Thus, the test participants were confused because of the fluctuation in vibration intensity that sometimes occurred during the test period. Such fluctuations might be problematic if a communication language is developed based on varying vibration intensities to indicate different information, as discussed by Cho and Park [59]. Therefore, it is suggested to use motors with lower fluctuation in rated speed. 

## 6. Conclusions

The reported construction worker fatalities and severe injuries indicate that construction sites are highly hazardous. To prevent such fatalities, it is important to identify potential hazards in advance and alert the concerned workers who are at risk. As such, researchers are continuously working to ameliorate the safety conditions of workers by the timely detection of possible hazards. However, there has not been significant progress in the second step, which involves alerting workers to take preventive actions. Thus, the authors conducted this study with the goal of developing a wearable system capable of alerting workers of risks in their surroundings. On construction sites, workers have to mainly rely on their senses of sight and hearing to perceive hazards. However, these senses are limited in the construction environment, and workers are often unable to identify risks. Therefore, in order to improve the hazard perception capability of construction workers, this study used a tactile-based system that allows workers to use the sense of touch on their backs to get warnings. This experimental study focused on determining a layout of tactile sensors that is suitable to transmit individualized hazard information to users. The authors identified 10 vibration motors to be sufficient to generate information related to hazards, and conducted a series of experiments to design the configuration of those ten motors. The experiments focused on configuring motors to result in higher accuracy of identifying signals from individual motors. Accordingly, the authors proposed a 4-2-4 configuration of motors (i.e., four motors on the top and bottom rows and two motors on the middle row) with all motors spaced at 3 inches.

This study proposed a wearable tactile sensory system for communicating hazard information to workers. While designing the layout, the authors focused on delivering three types of information: The direction of hazard point, type of hazard, and level of hazard for the individual worker. The test results, with a high accuracy of identifying signals from individual motors, indicate that the individual motors can be indexed to indicate particular information. Those indexed motors can be triggered in a sequence to deliver complete information to workers. However, the capability of this system to deliver multiple types of information has not been investigated in this study. Therefore, future studies will focus on investigating the effectiveness of the system to deliver multiple types of information to workers by the sequential vibration of the indexed motors. Further research will also focus on enabling this communication system to transmit tactile signals based on the data received from hazard detection systems. The application of such a wearable system, which does not affect the performance of construction workers, would play a significant role in improving the overall safety of the construction industry.

## Figures and Tables

**Table 1:** Spacing between motors (inch).

Spacing No.	Vertical Spacing (v)	Horizontal Spacing (h)
1	3.25	3.25
2	3.00	3.00
3	2.75	2.75
4	2.50	2.50
5	2.25	2.50
6	2.00	2.50

**Table 2:** Summary of perception accuracy in spacing study.

Spacing No.	Spacing between Motors (inch)		Accuracy of Identifying Individual Motors (%)					
	Vertical (v)	Horizontal (h)	M1	M2	M3	M4	M5	Overall
1	3.25	3.25	99.44	100	100	100	100	99.89
2	3.00	3.00	99.72	100	99.72	100	100	99.89
3	2.75	2.75	94.17	99.17	100	100	100	98.67
4	2.50	2.50	94.72	98.61	96.67	99.72	100	97.94
5	2.25	2.50	90.28	98.06	95.83	100	100	96.83
6	2.00	2.50	86.67	97.50	93.33	100	100	95.50
Overall Accuracy (%)			94.17	98.89	97.59	99.95	100	

**Table 3:** Signal misperceptions for motors: vertical axis.

	M1	M2	M3
**M1**	94.17%	5.83%	0.00%
**M2**	0.83%	99.03%	0.14%
**M3**	0.00%	2.36%	97.64%

**Table 4:** Signal misperceptions for motors: horizontal axis.

	M4	M2	M5
**M4**	99.95%	0.05%	0.00%
**M2**	0.05%	99.86%	0.09%
**M5**	0.00%	0.00%	100.00%

**Table 5:** Preliminary configuration test summary.

Test Participant	1-3-3-3 Configuration			4-2-4 Configuration		
	Minimum Accuracy	Maximum Accuracy	Average Accuracy	Minimum Accuracy	Maximum Accuracy	Average Accuracy
P1	82.50%	91.67%	87.17%	90.83%	96.67%	94.33%
P2	85.00%	89.17%	87.17%	93.33%	97.50%	95.83%
P3	73.33%	86.67%	81.17%	80.83%	96.67%	90.67%
P4	79.17%	92.50%	84.33%	90.83%	99.17%	95.50%
P5	73.33%	92.50%	85.33%	89.17%	99.17%	93.50%
P6	82.50%	94.17%	89.33%	89.17%	100.00%	96.67%

**Table 6:** Accuracy of correctly identifying individual signals.

Motors	M1	M2	M3	M4	M5	M6	M7	M8	M9	M10
Accuracy (%)	98.89	96.81	98.19	99.72	99.58	98.06	98.19	90.56	97.22	98.61
